# Precisely Navigated Biobot Swarms of Bacteria *Magnetospirillum magneticum* for Water Decontamination

**DOI:** 10.1021/acsami.2c16592

**Published:** 2023-01-26

**Authors:** Su-Jin Song, Carmen C. Mayorga-Martinez, Jan Vyskočil, Markéta Častorálová, Tomáš Ruml, Martin Pumera

**Affiliations:** † Center for Advanced Functional Nanorobots, Department of Inorganic Chemistry, Faculty of Chemical Technology, University of Chemistry and Technology Prague, Technická 5, Prague 166 28, Czech Republic; ‡ Department of Biochemistry and Microbiology, University of Chemistry and Technology Prague, Technická 5, Prague 166 28, Czech Republic; § Department of Chemical and Biomolecular Engineering, Yonsei University, 50 Yonsei-ro, Seodaemun-gu, Seoul 03722, Korea; ∥ Faculty of Electrical Engineering and Computer Science, VSB—Technical University of Ostrava, 17. listopadu 2172/15, Ostrava 70800, Czech Republic; ⊥ Department of Medical Research, China Medical University Hospital, China Medical University, No. 91 Hsueh-Shih Road, Taichung 40402, Taiwan

**Keywords:** magnetotactic bacteria, microrobots, nanorobots, magnetic actuation, micromotors

## Abstract

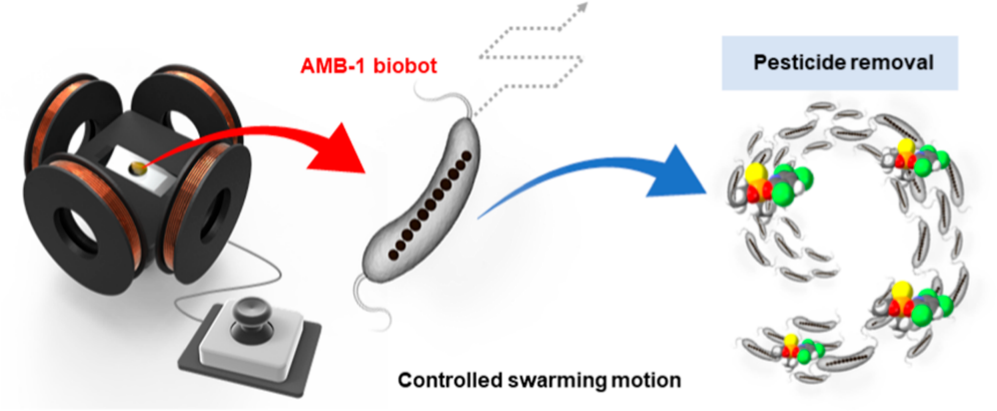

Hybrid biological robots (biobots) prepared from living cells are at the forefront of micro-/nanomotor research due to their biocompatibility and versatility toward multiple applications. However, their precise maneuverability is essential for practical applications. Magnetotactic bacteria are hybrid biobots that produce magnetosome magnetite crystals, which are more stable than synthesized magnetite and can orient along the direction of earth’s magnetic field. Herein, we used *Magnetospirillum magneticum* strain AMB-1 (*M. magneticum* AMB-1) for the effective removal of chlorpyrifos (an organophosphate pesticide) in various aqueous solutions by naturally binding with organic matter. Precision control of *M. magneticum* AMB-1 was achieved by applying a magnetic field. Under a programed clockwise magnetic field, *M. magneticum* AMB-1 exhibit swarm behavior and move in a circular direction. Consequently, we foresee that *M. magneticum* AMB-1 can be applied in various environments to remove and retrieve pollutants by directional control magnetic actuation.

## Introduction

Micro-/nanorobots driven by magnetic fields,^1−4^ light,^5,6^ and acoustic fields^7^ have made it possible to improve various investigations of targeted drug delivery,^8,9^ biosensing,^10−12^ and environmental remediation.^13−18^ In particular, magnetically driven biohybrid micro-/nanorobots are promising candidates in fields that require precise manipulation by integrating self-propulsion ability and magnetic actuation.^19−21^ Propulsion using a magnetic field has advantageous features such as remote control, fuel-free propulsion, and programmability.^22−25^ However, the integration of individual movements and the coherent orientation of the biohybrid micro-/nanorobots remains an important challenge.^26,27^

Magnetotactic bacteria (MTB), one of the natural species with magnetic properties, are Gram-negative prokaryotes and represent outstanding candidates as living micro-/nanorobots.^28^ They move along earth’s magnetic field by producing magnetosome magnetite crystals.^29,30^ Their innate magnetism allows them to swim farther than their body length per second and spontaneously form clusters, allowing for collective behaviors.^23,25^ In addition, the high motility of MTB can be actuated by combining with an external magnetic field.^23^ MTB have been used in various fields such as medicine,^31,32^ chemistry,^33^ physics,^34^ and biotechnology,^35^ either as whole cells or as extracted magnetosomes due to their magnetic properties.^36−38^ However, implementing MTB for the removal of environmental pollutants has not been sufficiently reported.^39^ Organophosphorus pesticides (*e.g.*, chlorpyrifos, parathion, and malathion), which have been revealed as one of the main causes of water pollution, introduce harmful effects on the surrounding environment and also on humans.^40−42^ Therefore, the study of pesticide detection and removal is essential to broaden our understanding. Ginet *et al.*(43) reported that the large surface areas of magnetic particles accelerated organic pollutant adsorption from the water environment; however, MTB present difficulties in terms of mass cultivation for application.^39^ To overcome this limitation, higher performance was demonstrated through studies that mimic natural swarming behavior.^44^

**Scheme 1:**
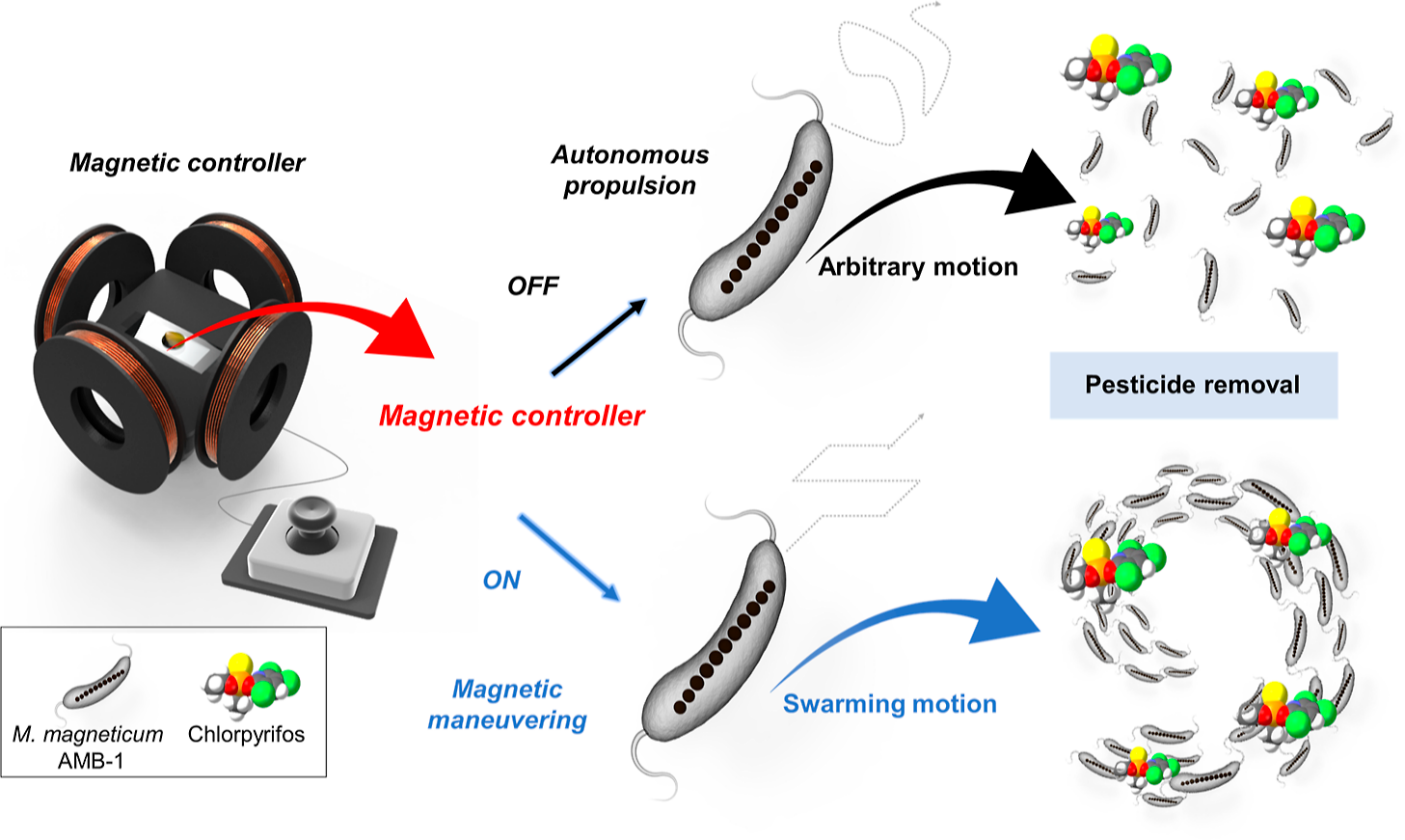
Schematic Diagram of *M. magneticum* AMB-1 Motion Behaviors for Pesticide Removal Maneuvering is achieved using a custom-made controllable magnetic field.

In this work, we present the *Magnetospirillum magneticum* strain AMB-1 (*M. magneticum* AMB-1), which combines autonomous self-propulsion and controlled magnetic maneuvering by a custom-made controllable magnetic field (Scheme 1). The innate magnetism of *M. magneticum* AMB-1 demonstrated their ability to control the motion by accurately depicting the letter “M”, “T”, and “B” using a joystick under the magnetic field. In addition, these bacteria play a role in biosorption through their movements, which mimic natural swarms when their self-propelling ability is combined with functional magnetic actuation. *M. magneticum* AMB-1 were placed into river aqueous solutions, allowing for precise directional control by modulating the input value of the applied rotating magnetic field under a programed clockwise automated mode. These results suggested that our *M. magneticum* AMB-1 biobots have attractive properties such as motility in various aqueous environments and a fast response to directional control by a controllable magnetic field. Therefore, we suggested that the precisely controllable *M. magneticum* AMB-1 biobot without additional manipulation such as surface modification or integration with functionalized nanoparticles are a promising candidate for inclusion in the biohybrid micro-/nanorobot field for aqueous pollutant removal.

## Results and Discussion

*M. magneticum* AMB-1 was cultured in a magnetic spirillum growth medium (MSGM) and produced magnetosome magnetite crystals. The cultivated *M. magneticum* AMB-1 growth was characterized using UV–vis spectroscopy, scanning electron microscopy (SEM), dark-field microscopy, transmission electron microscopy (TEM), and energy-dispersive X-ray spectroscopy (EDX). The custom-made controllable magnetic field was capable of maneuvering the *M. magneticum* AMB-1 in either a programed clockwise automated mode or in the desired direction using the manual mode. The precise mobility achieved is demonstrated by the letters created under the manual mode. Here, the efficiency of chlorpyrifos pesticide removal in river aquatic environments using the programed clockwise automated mode was investigated.

*M. magneticum* AMB-1 were cultured following a previously reported method^19^ (see the Experimental Section). Figure S1 shows the growth curve of *M. magneticum* AMB-1 measured at 565 nm (OD_565_) for 14 days. Sterile MSGM without cells was prepared for the control condition. The initial OD_565_ value of *M. magneticum* AMB-1 was approximately 0.04 ± 0.001 after re-suspension in a fresh medium, the same as the MSGM (blue dot, 0.04 ± 0.002).

**Figure 1:**
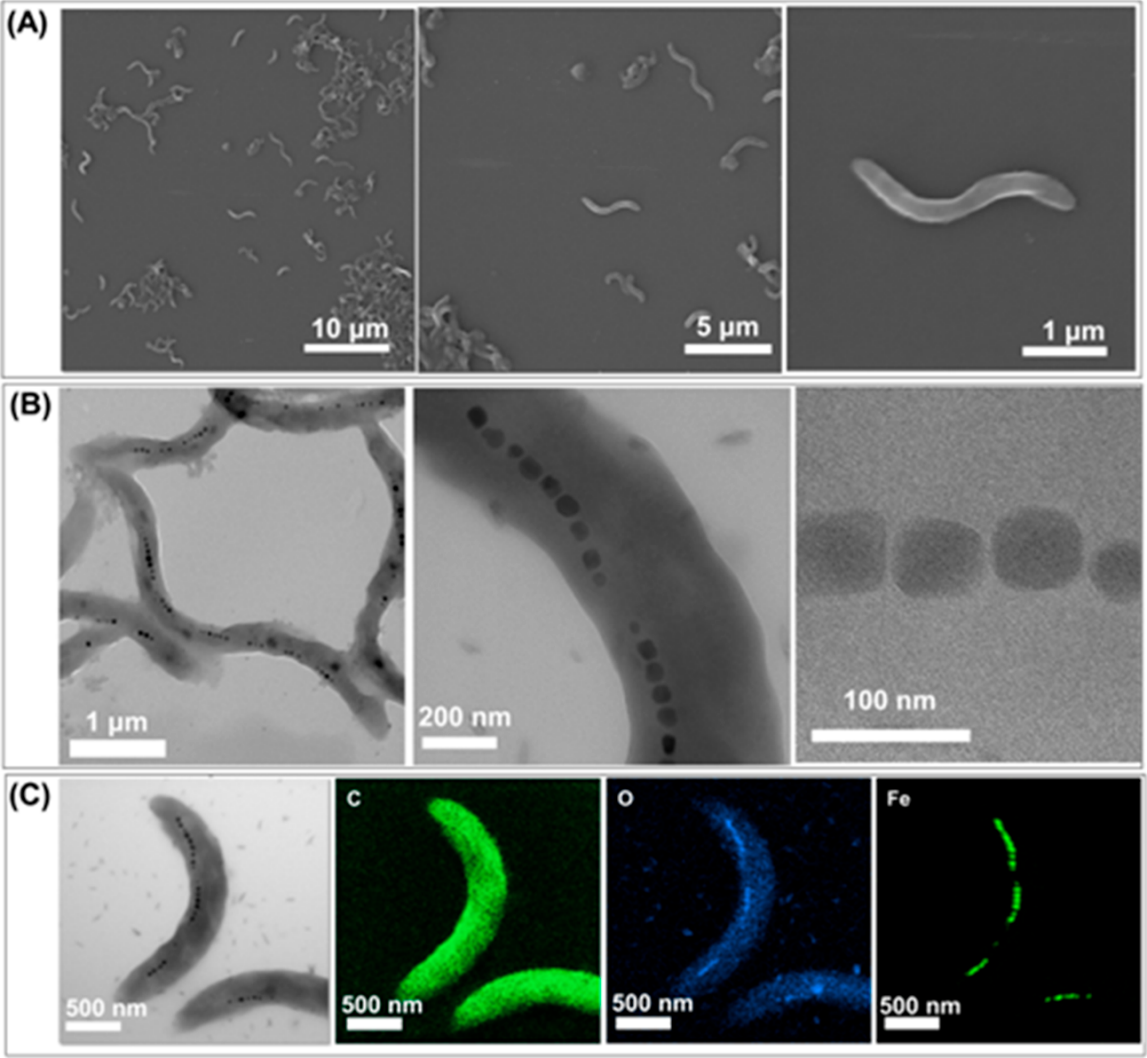
(A) SEM and (B) TEM images of *M. magneticum* AMB-1 at different magnifications. (C) EDX elementary mapping from TEM images of *M. magneticum* AMB-1.

To confirm the morphology and production of the biomineral in *M. magneticum* AMB-1, we carried out characterization by SEM, TEM, and EDX as well as dark-field microscopy. The SEM images at different magnifications (Figure 1A) show the morphology of *M. magneticum* AMB-1. The *M. magneticum* AMB-1 images reveal a spiral structure, and the average diameter and length were 0.39 ± 0.05 and 2.18 ± 0.43 μm, respectively. Moreover, TEM images at different magnifications as represented in Figure 1B demonstrate the formation of magnetosome chains inside *M. magneticum* AMB-1. Magnetosomes were homogeneously distributed within the cell and consisted of one to several chains with an average diameter of 41.65 ± 5.76 nm. In addition, EDX elemental mapping from the TEM image (Figure 1C) was used to verify the elements present in the magnetosomes. The corresponding results showed the elemental distribution of C, O, and Fe inside *M. magneticum* AMB-1, further indicating the existence of magnetite, which is arranged along the bacterial motion axis.^45^

Further, hyperspectral imaging from dark-field microscopy was performed to identify the location of the magnetosomes within *M. magneticum* AMB-1 (Figure S3). Hyperspectral imaging using the CytoViva technology as used in this study distinguishes the light reflected by the particles from the surroundings, enabling the recognition of particles. The visual evidence for the presence of particles produced within *M. magneticum* AMB-1 is shown in Figure 1B. From the representative dark-field image of *M. magneticum* AMB-1, aligned bright-colored particles were observed along the bacterial cell walls. In other words, external and internal regions of the cells can be distinguished through hyperspectral image analysis, and the corresponding spectral libraries can be collected from the pixels of each image. As shown in Figure S3A, the spectra revealed the conspicuous difference between the inside particle [1 of Figure S3A(i,ii)] and the background [2 of Figure S3A(i,ii)] of *M. magneticum* AMB-1; as observed, high reflectance was collected from the particles. In other words, this analysis assisted in confirming the presence of the particles.

**Figure 2:**
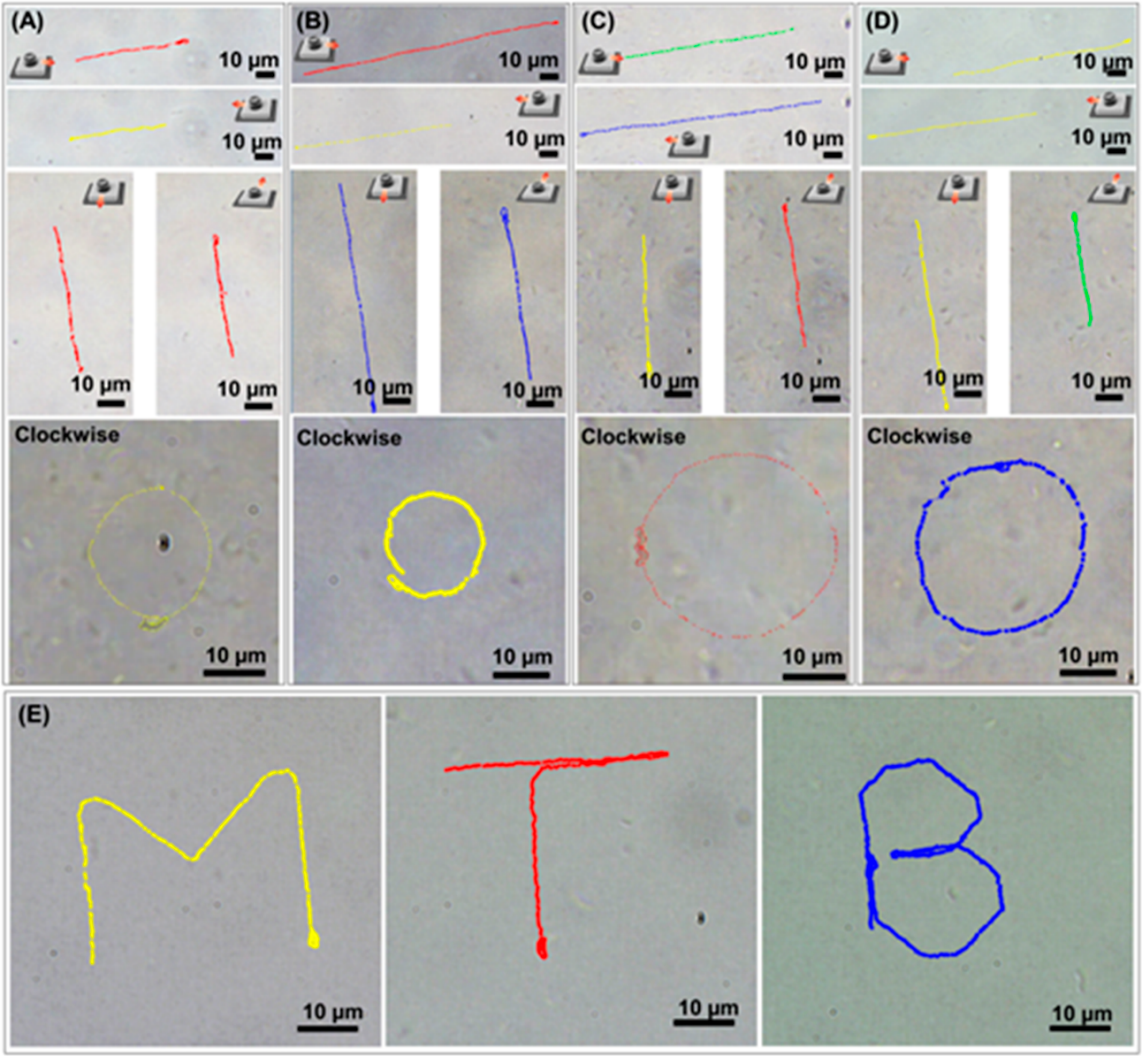
Digital images of swarm motion trajectories of *M. magneticum* AMB-1 in different directions: right, left, down, up, and clockwise. Experiments were performed in DW (A), medium (B), RW (C), and TW (D). Trajectories of directed *M. magneticum* AMB-1 to form the letters “M,” “T,” and “B” (E).

*M. magneticum* AMB-1 has helical structure flagella at either end of the cell, allowing it to swim forward or reverse directions through flagellar motion^23,24^ and swim along the earth’s magnetic field through their magnetosomes produced within the cell (Video S1).^46,47^ Therefore, precise direction control is essential to drive the bio-micro-/nanorobot through the interaction between *M. magneticum* AMB-1 innate magnetism and the external magnetic field.^48,49^ To visually investigate the dynamic control of *M. magneticum* AMB-1, the motion behaviors of cells were observed under the control of a custom-made rotating magnetic field produced by two pairs of coils fixed on an inverted microscope table and operated using a magnetic field controller (Figure S4). Controlling the direction of propulsion of *M. magneticum* AMB-1 using the custom-made controllable magnetic field is feasible due to the intracellular magnetite nanoparticles. Figure 2A–D shows captured images of *M. magneticum* AMB-1 propelled under 3 Hz and 5 mT using a joystick under the custom-made controllable magnetic field, where *M. magneticum* AMB-1 were driven in the maneuvering mode (right, left, down, and up) and autonomous mode (clockwise) in different aqueous solutions [distilled water (DW), river water (RW), and tap water (TW)]. In parallel, we also observed the maneuverability of *M. magneticum* AMB-1. Figure 2A–D shows representative tracking trajectories of *M. magneticum* AMB-1 driven under manual and autonomous modes in each solution. During the maneuvers, *M. magneticum* AMB-1 were propelled stably along the manipulated direction of the magnetic field in all aqueous solutions (Videos S2, S3, S4, S5, and S6).

The mean velocities of *M. magneticum* AMB-1 at different aqueous solutions were calculated, and the results are presented in Figure S5A. *M. magneticum* AMB-1 moved at speeds of 10 ± 3 μm/s (DW), 32 ± 5 μm/s (M), 19 ± 8 μm/s (RW), and 21 ± 4 μm/s (TW), respectively, under directional control. As a result, *M. magneticum* AMB-1 showed a different speed in each medium but did not affect motion control. Cells of the cultivated *Magnetospirillum* strains can align along magnetic field lines, but each cell does not display a preferred seeking polarity (north or south) and swims according to a magnetoreception mechanism.^47^ In addition, the MTB exposure to reducing conditions during microscopic analysis, or the characteristic “ping-pong” behavior, i.e., moving rapidly in the opposite direction of the applied magnetic field and returning slowly in the direction of the magnetic field, can affect the motility of the MTB.^50,51^ Moreover, the optical density (OD) values of the bacterial solutions before and after magnetic separation at variable times were measured (Figure S5B). An external magnet was positioned underneath the sample tube of each medium containing *M. magneticum* AMB-1, and the OD at 565 nm was measured after 10, 30, 60, and 180 min of magnetic separation. After 180 min, it was confirmed that the external magnet collected almost all *M. magneticum* AMB-1 in the aqueous solutions by decreasing the OD value. This experiment demonstrated that almost all MTB are active and responsive to external magnetic fields.

Besides, *M. magneticum* AMB-1 could be precisely maneuvered to form the letters “M,” “T,” and “B” using the manual mode (Figure 2E and Video S7). This experiment demonstrated the precise maneuvering of magnetic *M. magneticum* AMB-1 by the applied rotating magnetic field. It is important to point out here that almost all *M. magneticum* AMB-1 are synchronized and move in the same direction in a swarm-like manner. Bacteria without magnetosomes do not follow a swarm trajectory. Finally, no significant differences were observed in the motion performance of *M. magneticum* AMB-1 in all aqueous solutions used. The following experiment will be realized in RW as an example of a real-world application.

**Figure 3:**
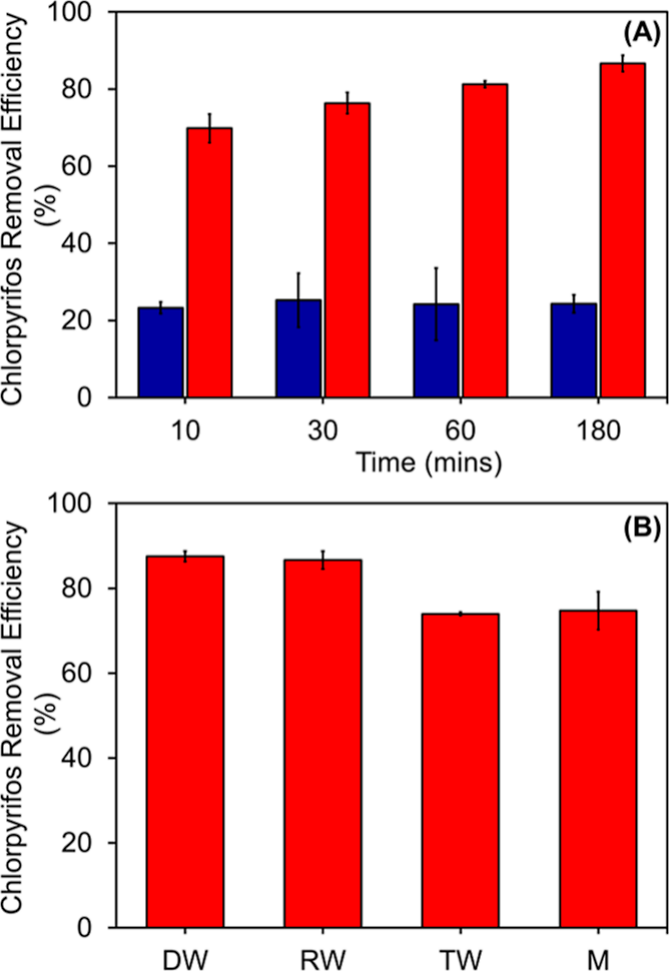
Chlorpyrifos removal efficiency of AMB-1 biobots (static: blue column and dynamic: red column) in RW (A). Comparison of the removal efficiency of chlorpyrifos in different aqueous solutions (DW—deionized water, RW—river water, TW—tap water, and M—medium) under the dynamic mode and after 180 min of magnetic actuation (B). Data are presented as the mean ± SD, and the experiment was repeated with *n* = 4.

MTB can remove water pollutants through their ability to naturally bind with organic matter.^52,53^ To evaluate the ability of AMB-1 biobots (we call AMB-1 biobots during the *M. magneticum* AMB-1 remove pesticides) for water pollutant remediation), we investigated the pesticide chlorpyrifos in RW to measure removal efficiency in static and dynamic (autonomous; circle) modes for 180 min (Figure 3A). RW was selected to test the real-world application of AMB-1 biobots. Within the first 10 min, more than 70% of chlorpyrifos had been removed under the dynamic mode. However, chlorpyrifos removal efficiency reached 76, 81, and 86% after 30, 60, and 180 min, respectively. In comparison, more than 70% of chlorpyrifos was retained in the solutions under the static mode experiment at the initial time. Nevertheless, no significant improvements in the pesticide removal efficiency over time were observed in the static mode. The AMB-1 biobots propelled to swarm in the dynamic mode demonstrated higher removal efficiency than in the static mode. For the experiments in the static mode, AMB-1 biobots were centrifuged, and the pellet (fraction remaining in the tube bottom) was stored using an external magnet.

Chlorpyrifos removal by AMB-1 biobots was performed for 180 min. The final removal percentages under the dynamic mode from deionized water (DW), RW, TW, and medium were 87.5, 86.6, 73.9, and 74.7%, respectively, as shown in Figure 3B. AMB-1 biobots achieved the highest removal rate in DW and RW.

## Conclusions

In this study, we demonstrated that MTB can be turned into biomicrorobots *via* magnetic actuation. These biodegradable robots can effectively remove the chlorpyrifos pesticide—one of the most widely used organophosphate pesticides in agriculture—from various water conditions. *M. magneticum* AMB-1 can successfully swim and thrive in media without any additional supplemental materials. Consequently, MTB can be motion-controlled under a custom-designed programed rotating magnetic field through the magnetosomes synthesized naturally by them during the incubation period. Moreover, because *M. magneticum* AMB-1 can move in groups in the same direction *via* directional manipulation, micromixing occurs, which increases pesticide removal efficiency. The controllable *M. magneticum* AMB-1 reported here can be propelled under a magnetic field and can efficiently remove pesticides on-the-fly. In the same vein, *M. magneticum* AMB-1 can be suggested as a new candidate for biohybrid active materials for biomedical and environmental applications. This work is limited to lab-scale experiments demonstration, but we also consider their real-world application in the future. One possible approach for this could be the use of an automated magnetic field setup, placed on a water treatment plant tube, following by using a permanent magnet for the poisoned MTB retrieval.

## Experimental Section

### Culture and Growth of *M. magneticum* AMB-1

*M. magneticum* AMB-1 (ATCC 700264) was grown at 30 °C in 50 mL of the MSGM containing the following ingredients (values in grams per liter): 0.68 g of potassium dihydrogen phosphate (KH_2_PO_4_), 0.85 g of succinic acid, 0.57 g of tartaric acid, 0.083 g of sodium acetate, and 0.17 g of sodium nitrate (NaNO_3_), supplemented with 10 mL of Wolfe’s vitamin solution, 5 mL of Wolfe’s mineral solution, and 2 mL of 0.01 M ferric quinate (0.27 g of FeCl_3_ and 0.19 g of quinic acid in 100 mL of DW). The final pH of MSGM was adjusted to 6.75 before sterilization. A microaerobic condition was set up by injecting the MSGM with nitrogen (N_2_) gas for 10 min. *M. magneticum* AMB-1 growth was evaluated via the OD at 565 nm (OD_565_) using a plate reader for 14 days.^54−56^ To clarify the proliferation of magnetic *M. magneticum* AMB-1, we observed the color change of the MSGM after adding 0.1% resazurin, which is used as an oxidation–reduction indicator. The resazurin changes from pink in its oxidized form to colorless when it is reduced and does not affect the proliferation of *M. magneticum* AMB-1.^57,58^ The MSGM with *M. magneticum* AMB-1 changed to colorless, whereas without *M. magneticum* AMB-1, the color remained pink, suggesting that *M. magneticum* AMB-1 proliferated by consuming oxygen during the culture period (Figure S2). Moreover, after 3 days of incubation, a dark-colored cluster was detected on the bottom of the tube; presumably, these are magnetosomes produced inside *M. magneticum* AMB-1.^59^ In the same vein, it could be explained that *M. magneticum* AMB-1 were proliferated and magnetosomes produced because microaerobic conditions were maintained during the culture period.^49^ Furthermore, *M. magneticum* AMB-1 was separated from each aqueous solution using an external magnet, and the OD value was measured at 565 nm at variable times.

### Characterization

Morphologies of *M. magneticum* AMB-1 and magnetosome crystals were analyzed using SEM, affiliated EDX, TEM, and hyperspectral microscopy analysis. The cells were obtained by centrifugation at 5939 RCF for 10 min. The supernatant was discarded and fixed in 5% glutaraldehyde in 0.1 M phosphate-buffered saline (pH 7.2) for 1 h at room temperature. Afterward, the cells were washed with DW and dehydrated in an ethanol series (40 to 100%).

### Motion Study

The motion of *M. magneticum* AMB-1 was controlled in different aqueous media: *M. magneticum* AMB-1 growth medium (ATCC medium 1653), DW, TW, and RW from the Vltava river. *M. magneticum* AMB-1 were centrifuged at 5939 RCF for 10 min and then transferred to each medium. A 3D-printed coil holder was designed and produced to fit two pairs of coils and placed on an inverted microscope table (Figure S4). The rotating magnetic field was created by a power supply with two current sources, with an exact phase shift of π/2 between the currents and variable frequency and amplitude. Motion was recorded using a 100× optical Nikon microscope (Eclipse Ts2), and the tracked trajectories of *M. magneticum* AMB-1 were analyzed on the recorded video using NIS Elements AR 3.2 software. The speed, which depended on the direction of *M. magneticum* AMB-1, was also analyzed using the NIS element tracking module.

### Removal of Chlorpyrifos

To investigate chlorpyrifos removal efficiency by AMB-1 biobots, cells were centrifuged (5939 RCF, 10 min) and re-suspended in different media: growth medium, DW, TW, and RW. The chlorpyrifos solution of 10 ppm was added to the AMB-1 biobots (density of 1.0 × 10^8^ cells/mL) using the rotation mode of the controllable magnetic field at room temperature. The chlorpyrifos removal efficiency of AMB-1 biobots was assessed for 180 min (*n* = 4). The statics of AMB-1 biobots was demonstrated under the same method except for the magnetic field. After the chlorpyrifos removal process, dynamic groups were centrifuged at 5939 RCF for 10 min, and all groups were quantified by UV–vis spectroscopy, referring to other previous studies.^60−62^ The different concentrations of chlorpyrifos have also been measured by UV–vis spectroscopy (Figure S6). The removal efficiency of chlorpyrifos (%) was calculated using eq 1
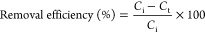
1where *C*_i_ is the initial concentration of chlorpyrifos in solution and *C*_t_ is the final concentration of chlorpyrifos solution after removal.

## Supporting Information Available

Growth curves of *M. magneticum* AMB-1 in culture for 14 days; cultivation of *M. magneticum* AMB-1 with resazurin; hyperspectral images of *M. magneticum* AMB-1; custom-made controllable magnetic field using a joystick; average velocity and magnetic separation of *M. magneticum* AMB-1 in different solutions; and UV–vis absorption spectra of chlorpyrifos (PDF)
Arbitrary motion of the MTB biobot in different aqueous solutions (MP4)
Motion study of MTB biobot in DW (MP4)
Motion control of the MTB biobot in the medium (MP4)
Motion control of MTB in RW (MP4)
Motion control of the MTB biobot in TW (MP4)
Motion control of the MTB biobot in different aqueous solutions (clockwise motion) (MP4)
Letter trajectory of the MTB biobot (MP4)
