# Supplementary material for: Precisely Navigated Biobot Swarms of Bacteria Magnetospirillum magneticum for Water Decontamination
Source: ACS Appl Mater Interfaces. 2023 Jan 26;15(5):7023–9. doi: 10.1021/acsami.2c16592 (PMC10016748; doi:10.1021/acsami.2c16592)
Supplement: Supplementary file 1 — am2c16592_si_001.pdf [file am2c16592_si_001.pdf]

Supporting Information

Precisely Navigated Biobot Swarms of Bacteria *M. magneticum* for Water Decontamination

Su-Jin Song,[1] Carmen C. Mayorga-Martinez,[1] Jan Vyskočil,[1] Markéta Častorálová,[2] Tomáš Ruml,[2] Martin Pumera[1,3,4,5*]

[1]Center for Advanced Functional Nanorobots, Department of Inorganic Chemistry, Faculty of Chemical Technology, University of Chemistry and Technology Prague, Technická 5, Prague 166 28, Czech Republic

[2]Department of Biochemistry and Microbiology University of Chemistry and Technology Prague, Technická 5, Prague 166 28, Czech Republic

[3]Department of Chemical and Biomolecular Engineering, Yonsei University, 50 Yonsei-ro, Seodaemun-gu, Seoul 03722, Korea

[4]Faculty of Electrical Engineering and Computer Science, VSB - Technical University of Ostrava, 17. listopadu 2172/15, 70800 Ostrava, Czech Republic

[5]Department of Medical Research, China Medical University Hospital, China Medical University, No. 91 Hsueh-Shih Road, Taichung 40402, Taiwan

*Corresponding author: pumera.research@gmail.com

TABLE OF CONTENTS

Figure S1. Growth curves of *M. magneticum* AMB-1 in culture for 14 days using the magnetic spirillum growth medium (MSGM).

Figure S2. Cultivation of *M. magneticum* AMB-1 in MSGM with resazurin. To identify *M. magneticum* AMB-1 that has grown by consuming oxygen during the incubation period. The blank control was pink color MSGM without *M. magneticum* AMB-1.

Figure S3. (A) Hyperspectral imaging of *M. magneticum* AMB-1. (B) Scattering spectra from pixels in the magnified images (A-i) and (A-ii).

Figure S4. Custom-made controllable magnetic field with joystick. Two pairs of coils are mounted on the microscopy to actuate the *M. magneticum* AMB-1.

Figure S5. (A) Average velocity of *M. magneticum* AMB-1 under directional control in different aqueous solution. (B) OD value comparison after magnetic separation of *M. magneticum* AMB-1.

Figure S6. UV-vis absorption spectra of chlorpyrifos. (A) UV–vis absorption spectra (0 ppm to 100 ppm) and (B) linear calibration plots for Chlorpyrifos.

1. **Video S1.** Arbitrary motion of MTB-BioBot in different aqueous solution
2. **Video S2.** Motion study of MTB-BioBot in DW
3. **Video S3.** Motion control of MTB-BioBot in Medium
4. **Video S4.** Motion control of MTB in River water
5. **Video S5.** Motion control of MTB-BioBot in Tap water
6. **Video S6.** Motion control of MTB-BioBot in different aqueous solution (Clockwise motion)
7. **Video S7.** Letter trajectory of MTB-BioBot

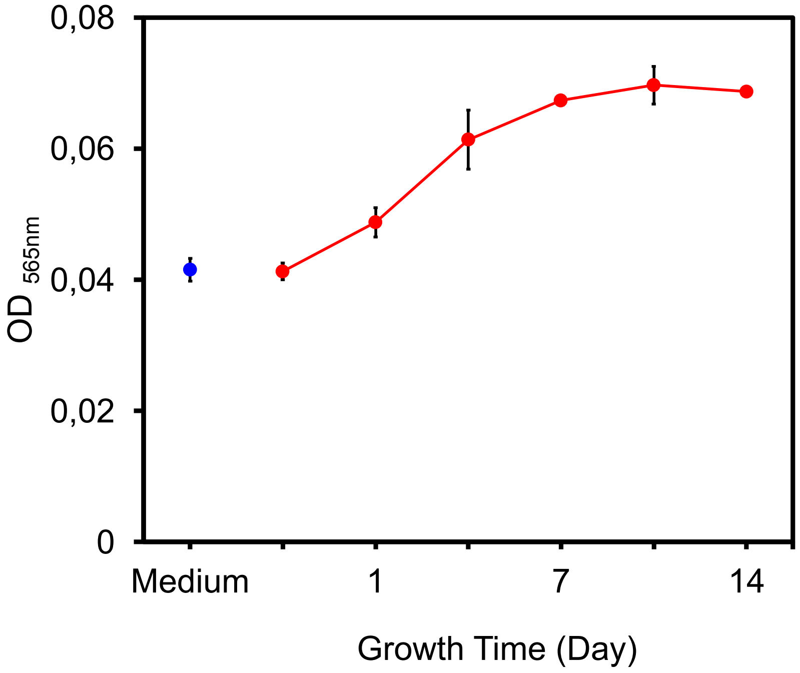

Figure S1. Growth curves of *M. magneticum* AMB-1 in culture for 14 days using the magnetic spirillum growth medium (MSGM).

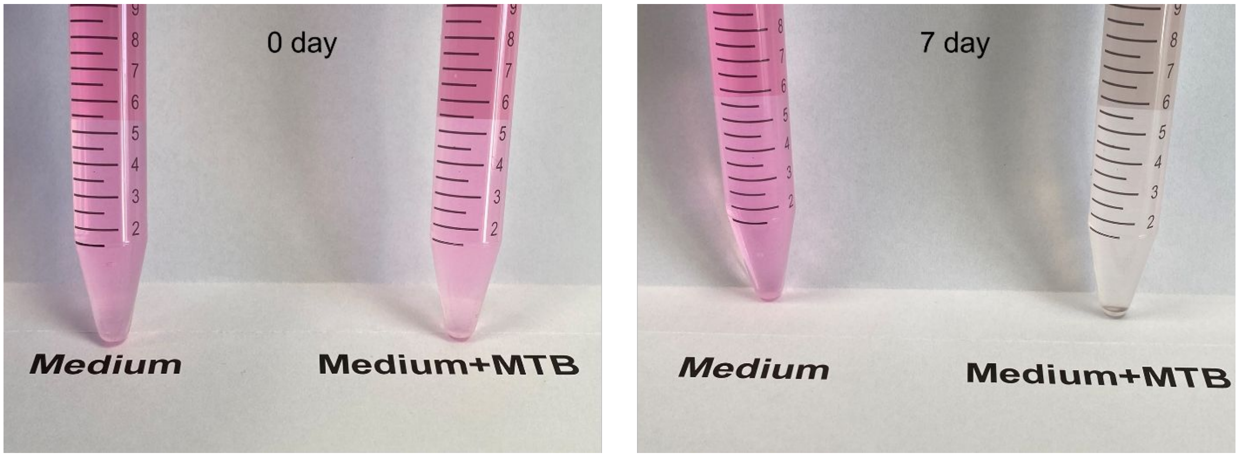

Figure S2. Cultivation of *M. magneticum* AMB-1 in MSGM with resazurin. To identify *M. magneticum* AMB-1 that has grown by consuming oxygen during the incubation period. The blank control was pink color MSGM without *M. magneticum* AMB-1.

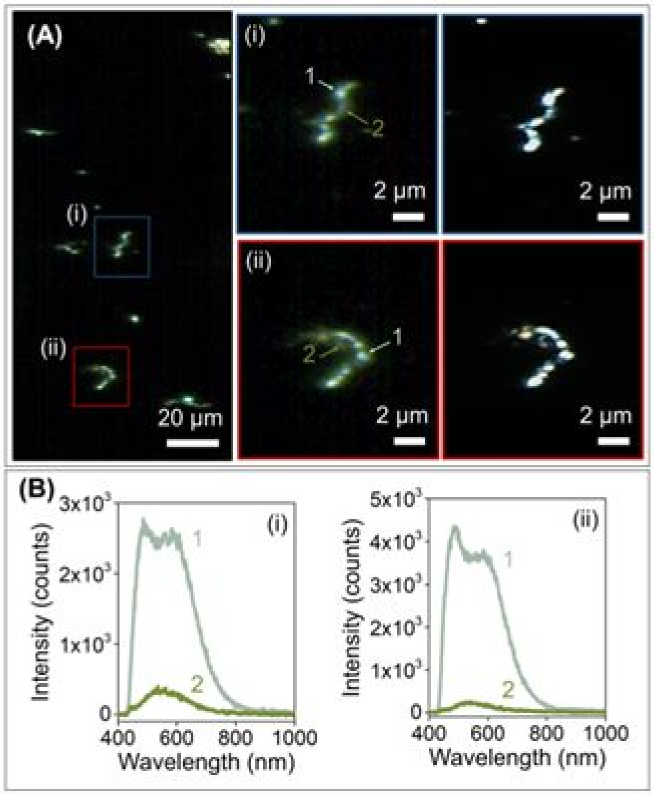

Figure S3. (A) Hyperspectral imaging of *M. magneticum* AMB-1. (B) Scattering spectra from pixels in the magnified images (A-i) and (A-ii).

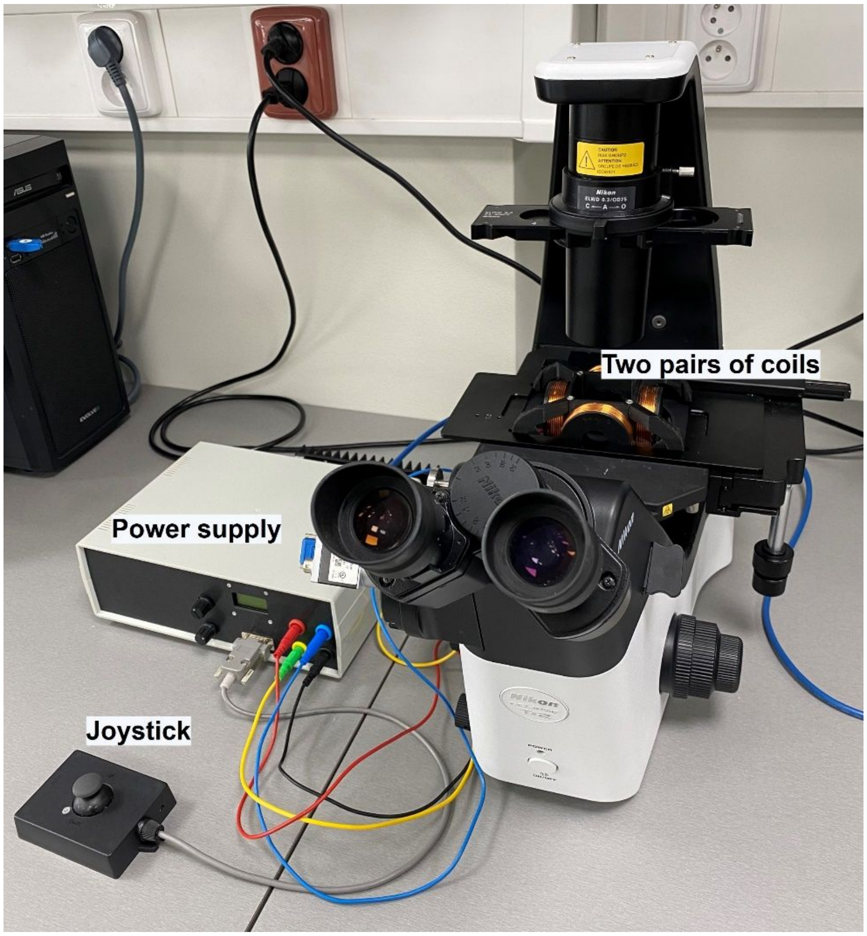

Figure S4. Custom-made controllable magnetic field with joystick. Two pairs of coils are mounted on the microscopy to actuate the *M. magneticum* AMB-1.

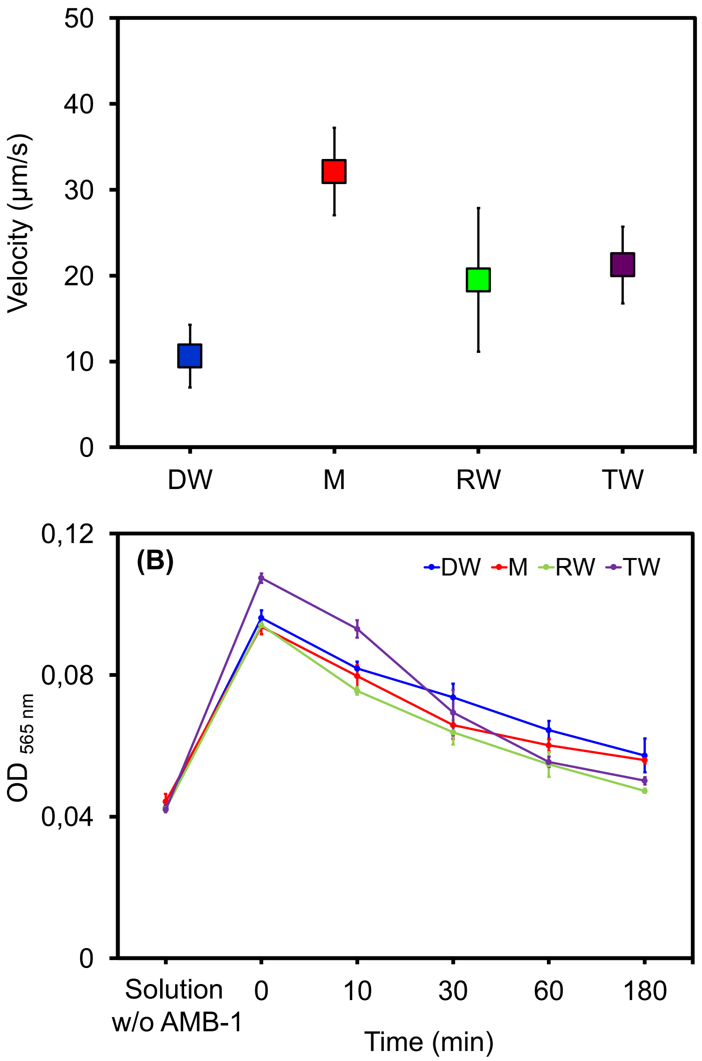

Figure S5. (A) Average velocity of *M. magneticum* AMB-1 under directional control in different aqueous solution. (B) OD value comparison after magnetic separation of *M. magneticum* AMB-1. (Solution; DW, M, RW, TW)

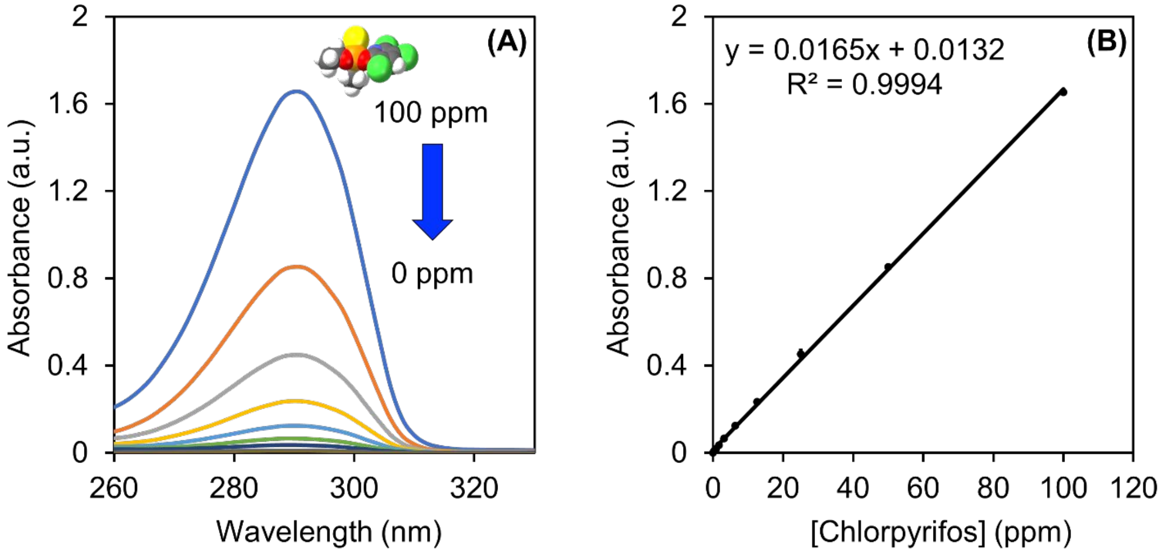

Figure S6. UV-vis absorption spectra of chlorpyrifos. (A) UV–vis absorption spectra (0 ppm to 100 ppm) and (B) linear calibration plots for Chlorpyrifos.
